# A Diagnostic and Therapeutic Approach to Retained Anchoring Sutures: Color Doppler Ultrasound for Diagnosing and the Retract-and-Cut Technique for Minimizing Invasive Interventions

**DOI:** 10.1155/carm/3019138

**Published:** 2025-04-07

**Authors:** Ali Hajihashemi, Reza Tavakoli, Mahsa Geravandi

**Affiliations:** ^1^Department of Radiology, School of Medicine, Isfahan University of Medical Sciences, Isfahan, Iran; ^2^Department of Radiology, Arak University of Medical Sciences, Arak, Iran

**Keywords:** catheter-related complication, color Doppler ultrasound, pigtail catheter complication, retained suture, retract-and-cut technique

## Abstract

**Background:** Retained sutures following catheterization procedures are rare but can present significant diagnostic and therapeutic challenges. This case highlights a novel approach to diagnosing and managing a retained anchoring suture following catheter removal for abdominal fluid drainage in a patient with pancreatic adenocarcinoma and metastatic ascites. The use of color Doppler ultrasound to identify the suture's path and the application of the retract-and-cut technique minimized invasive interventions, demonstrating a safe and effective alternative to surgical removal.

**Case Presentation:** A 68-year-old male with pancreatic adenocarcinoma and metastatic ascites underwent subhepatic fluid drainage using an 8Fr pigtail catheter. After successful drainage and catheter removal, the patient presented with localized pain and tenderness at the catheter insertion site. Ultrasound revealed a hyperechoic linear structure in the subcutaneous tissue suggestive of a retained suture. Real-time visualization using color Doppler ultrasound confirmed the suture's path as a linear Doppler signal was observed during manipulation. Given the adhesion of the suture to deeper tissues, the retract-and-cut technique was employed. The suture was gently pulled taut at the skin surface, cut, and allowed to retract along its original track, avoiding unnecessary trauma. The patient experienced no recurrence of symptoms, fluid collection, or infection during long-term follow-up.

**Conclusions:** This case underscores the importance of timely diagnosis using color Doppler ultrasound, which provided real-time visualization of the retained suture and its relationship with surrounding tissues. In addition, the retract-and-cut technique offers a minimally invasive and effective approach for managing retained sutures, avoiding the need for surgical intervention. This method ensures patient comfort and safety, particularly in palliative care settings where nonsurgical options are prioritized.

## 1. Introduction

Retained sutures following catheterization procedures is a rare but important complication that can pose diagnostic and therapeutic challenges [1, 2]. This case discusses the diagnosis and management of a complication resulting from a retained anchoring suture after catheter drainage for an abdominal fluid collection in a patient with pancreatic adenocarcinoma and metastatic ascites. While catheterization for fluid drainage is common in palliative care, the retained suture posed an unusual complication. The use of color Doppler ultrasound, an imaging tool, helped identify the retained suture's trajectory, which is key to ensuring precise and minimally invasive removal. This case highlights that the complication does not always require invasive surgery for suture removal and that conservative techniques, such as the retract-and-cut method used in this case, can be effective and safe in the long term. The successful outcome in this patient underscores the potential for nonsurgical approaches in managing similar complications, with favorable long-term results.

## 2. Case Presentation

A 68-year-old male with a history of pancreatic adenocarcinoma with metastatic disease and associated ascites was admitted for management of an abdominal fluid collection. Given his poor prognosis and persistent fluid accumulation, an 8Fr nephrostomy catheter, a type of pigtail catheter, was inserted to drain the subhepatic fluid collection. The patient was placed on broad-spectrum antibiotics for the duration of catheter placement to prevent infection.

After an 8-week period, a follow-up ultrasound demonstrated that the fluid collection had resolved with minimal drainage output, indicating successful drainage and resolution of the underlying collection. Upon catheter removal, the patient initially showed signs of clinical improvement. However, a few days later, he developed localized pain and tenderness at the catheter removal site. A repeat ultrasound was performed to investigate the source of the pain. The imaging revealed a hyperechoic linear structure in the subcutaneous tissue at the catheter insertion site, with no evidence of the suture path at deeper levels. No evidence of collection/abscess was noted at this site.

**Figure 1:**
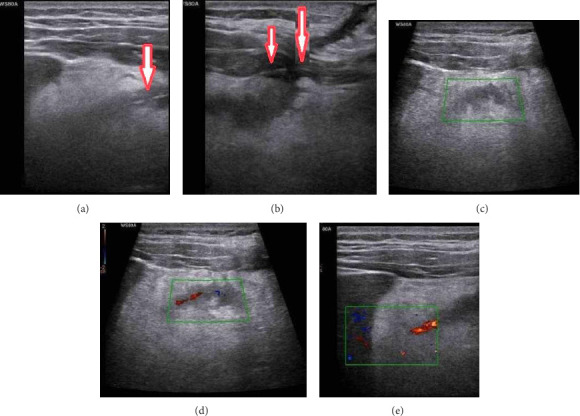
Gray-scale ultrasonography revealed a hyperechoic line at the superficial aspect of the catheter track, with no evidence of the suture path at deeper levels (a-b, red arrows). Color Doppler ultrasound was then used. Although the color Doppler was active, no signal was observed as the suture had not been manipulated (c). After manipulating the suture, a linear Doppler signal was observed, corresponding to the path of the manipulated suture (d-e). The pulse repetition frequency and Doppler settings used were G50/0.40 kHz/F1/FA8, with the flow velocity estimated to be within the expected physiological range for superficial soft tissue perfusion.

Upon identifying a clue on the surface (skin), a small incision was made under sterile conditions after disinfecting the area with chlorhexidine. The tip of the retained suture was visible in the superficial soft tissue. Color Doppler ultrasound was activated. A low pulse repetition frequency (PRF) setting was used to avoid aliasing and to accurately visualize the suture's path. This also helped to exclude any local hypervascularization in the area surrounding the retained suture, ensuring that no abnormal blood flow was detected. The suture was gently manipulated by moving it back and forth, and a color Doppler signal appeared linearly as the suture moved (Figure 1, Video 1).

This confirmed the presence and path of the retained anchoring suture, likely used to secure the catheter during the drainage procedure. This provided real-time information about the suture's relationship with surrounding tissues and vasculature, confirming its position and course within the patient's body tissues.

Once the suture's location was confirmed, it was gently manipulated (pulled outward); however, due to adhesions to the deeper tissue, the suture could not be removed. Therefore, the retract-and-cut technique was selected for removal. The first step involved gently pulling the suture taut at the surface of the skin over the catheter track. This ensured the suture was under tension and could be retracted back into the tissue once it was cut, minimizing the amount of retained suture left in the tissue.

The suture was then carefully cut at the skin surface. This allowed the suture to retract along its original track. Cutting the suture in this manner avoids unnecessary trauma to surrounding tissues, as the suture is pulled back into its original pathway rather than being forcibly removed.

Following the retraction and cutting of the suture, the patient was closely observed for any recurrence of fluid collection, infection, or signs of inflammation at the site of the retained suture. During the long-term follow-up period, the patient reported no further symptoms or complaints. Follow-up imaging, including ultrasound, was conducted to confirm complete resolution of the issue. No complications, such as infection, were noted.

## 3. Discussion

Pigtail catheters are maintained straight during insertion, and upon deployment are curled into an annular shape. These catheters are especially useful for the drainage of abscesses or of the urinary system. The pigtail construction promotes retention of the catheter in the target location (e.g., within the collection) [3]. The distal tips of conventional pigtail catheters are looped by pulling a string or suture extending from the distal tip along the catheter to a manual control at the proximal end and the catheter is then connected to another device (e.g., a collection bag and additional tubing) using, for example, a pair of locking hubs. The occurrence of a retained anchoring suture following catheter placement is rare and underreported. The suture(string) inside the pigtail catheter serves to create a loop at the tip of the catheter, which helps secure the catheter in place within the tissue but if they remain embedded or improperly anchored, they can lead to discomfort, inflammation, or further complications [3] This case illustrates the diagnostic challenges associated with retained sutures, where imaging findings may not always provide a diagnostic image, especially if the suture is not easily visible at deeper levels. In this patient, the use of color Doppler ultrasound played a crucial role in confirming the presence of the retained suture. The Doppler signal, which aligned with the trajectory of the suture, provided real-time visualization of the suture's path through surrounding tissues. This is a key advancement and novel approach in diagnostic imaging, offering a noninvasive method for localizing and tracking the suture's location, which could have, otherwise, been missed in conventional imaging. It is important to note that color Doppler ultrasound, beyond its traditional use in assessing blood flow, can be particularly useful for identifying foreign bodies like sutures. In this case, color Doppler was employed to visualize the retained anchoring suture. As the suture was gently manipulated by moving it back and forth, a color Doppler signal appeared linearly, corresponding to the suture's movement. This real-time imaging technique allowed for precise tracking of the suture's location, confirming both its presence and its path within the tissues. Furthermore, the Doppler signal provided valuable information about the suture's relationship with surrounding vasculature and tissues. This approach offered a clear advantage in identifying and confirming the suture's trajectory, something that might have been difficult to detect with conventional imaging alone.

Another important aspect of this case is the Therapeutic approach. While surgical intervention might traditionally be considered for such retained sutures, this case demonstrates that a nonsurgical method—specifically the retract-and-cut technique—can be just as effective [4]. Upon reviewing the existing literature, there is limited documentation regarding the management of retained anchoring sutures following catheter procedures. In one such study, the authors reported that in their experience, the anchoring suture could often be extracted through gentle manipulation with a dilator. However, this method was not always successful. In fact, the authors encountered 12 cases where this approach failed, necessitating the use of alternative strategies.

One of the methods introduced in the paper was the retract-and-cut method, which was presented as a reliable solution for cases in which the anchoring suture becomes adherent to the surrounding tissue. This technique, similar to the one used in our case, involves carefully cutting the suture at the surface level and retracting it along its original path, minimizing damage to surrounding tissues and avoiding the need for invasive surgery. The retract-and-cut method was shown to be effective in those instances where other methods, like dilator manipulation, were not feasible.

By gently manipulating (pulling outward) the suture and cutting it, unnecessary trauma to surrounding tissues was avoided. This approach ensured a more comfortable and less invasive treatment. This technique not only minimizes surgical risk but also promotes faster recovery, with no complications or infections observed during follow-up. The long-term absence of infection in this case further supports the safety and efficacy of this approach. Although retained sutures are rare, this case shows that they can be managed effectively with noninvasive techniques and careful monitoring, suggesting that surgical removal is not always necessary. The patient's successful recovery highlights the viability of less invasive treatment options in managing catheter-related complications, which may be particularly relevant in palliative or end-of-life care where minimizing patient discomfort and risk is essential [4–7].

In conclusion, the case emphasizes the importance of timely and accurate diagnosis using advanced imaging techniques like color Doppler ultrasound, and it advocates for conservative treatment options in the management of retained sutures. The technique used in this case represents a safe, noninvasive solution with positive long-term outcomes, providing an alternative to more invasive surgical procedures. This approach can be considered in similar cases where a retained suture is suspected, improving patient care and outcomes in the management of such complications.

## Data Availability

The data that support the findings of this study are available from the corresponding author upon reasonable request.

## Supporting Information

The Supporting Information includes two ultrasound videos that complement the findings presented in the main manuscript. These videos provide additional details of the ultrasound observations and offer a clearer understanding of the results described in the text.

Video 1 (A, B): After manipulating the suture (string), a linear Doppler signal was observed, corresponding to the path of the manipulated suture.
